# Mass spectrometry-based analysis of eccrine sweat supports predictive, preventive and personalised medicine in a cohort of breast cancer patients in Austria

**DOI:** 10.1007/s13167-025-00396-6

**Published:** 2025-01-31

**Authors:** Michael Bolliger, Daniel Wasinger, Julia Brunmair, Gerhard Hagn, Michael Wolf, Karin Preindl, Birgit Reiter, Andrea Bileck, Christopher Gerner, Florian Fitzal, Samuel M. Meier-Menches

**Affiliations:** 1 Department of General Surgery (Division of Visceral Surgery), Medical University of Vienna, Waehringer Guertel 18-20, 1090 Vienna, Austria; 2 Department of Surgery, St. Francis Hospital, Nikolsdorfergasse 32, 1050 Vienna, Austria; 3 Faculty of Chemistry, Department of Analytical Chemistry, University of Vienna, Waehringer Str. 38, 1090 Vienna, Austria; 4 Vienna Doctoral School in Chemistry, University of Vienna, Waehringer Str. 38-42, 1090 Vienna, Austria; 5 Department of Laboratory Medicine, Medical University of Vienna, Waehringer Guertel 18–20, Vienna, 1090 Austria; 6 Joint Metabolome Facility, University of Vienna and Medical University Vienna, Waehringer Str. 38, 1090 Vienna, Austria; 7 Department of Surgery and Vascular Surgery, Hanusch Hospital, Heinrich-Collin-Str. 30, 1140 Vienna, Austria; 8 Faculty of Chemistry, Institute of Inorganic Chemistry, University of Vienna, Waehringer Str. 38, 1090 Vienna, Austria

**Keywords:** Breast cancer, Lifestyle parameters, Systemic response to therapy, Eccrine sweat, Metabolomics, Patient stratification, Risk assessment, Disease progression, Evidence-based research data, Individualised patient monitoring, Molecular patterns, Multi-omics, Predictive preventive personalised medicine (PPPM / 3PM), Secondary tertiary care, Sweat metabotyping

## Abstract

**Objective:**

Metabolomics measurements of eccrine sweat may provide novel and relevant biomedical information to support predictive, preventive and personalised medicine (3PM). However, only limited data is available regarding metabolic alterations accompanying chemotherapy of breast cancer patients related to residual cancer burden (RCB) or therapy response. Here, we have applied Metabo-Tip, a non-invasive metabolomics assay based on the analysis of eccrine sweat from the fingertips, to investigate the feasibility of such an approach, especially with respect to drug monitoring, assessing lifestyle parameters and stratification of breast cancer patients.

**Methods:**

Eccrine sweat samples were collected from breast cancer patients (*n* = 9) during the first cycle of neoadjuvant chemotherapy at four time points in this proof-of-concept study at a Tertiary University Hospital. Metabolites in eccrine sweat were analysed using mass spectrometry. Blood plasma samples from the same timepoints were also collected and analysed using a validated targeted metabolomics kit, in addition to proteomics and fatty acids/oxylipin analysis.

**Results:**

A total of 247 exogenous small molecules and endogenous metabolites were identified in eccrine sweat of the breast cancer patients. Cyclophosphamide and ondansetron were successfully detected and monitored in eccrine sweat of individual patients and accurately reflected the administration schedule. The non-essential amino acids asparagine, serine and proline, as well as ornithine were significantly regulated in eccrine sweat and blood plasma over the therapy cycle. However, their distinct time-dependent profiles indicated compartment-specific distributions. Indeed, the metabolite composition of eccrine sweat seems to largely resemble the composition of the interstitial fluid. Plasma proteins and fatty acids/oxylipins were not affected by the first treatment cycle. Individual smoking habit was revealed by the simultaneous detection of nicotine and its primary metabolite cotinine in eccrine sweat. Stratification according to RCB revealed pronounced differences in the metabolic composition of eccrine sweat in these patients at baseline, e.g., essential amino acids, possibly due to the systemic contribution of breast cancer and its impact on metabolic turnover.

**Conclusion:**

Mass spectrometry-based analysis of metabolites from eccrine sweat of breast cancer patients successfully qualified lifestyle parameters for risk assessment and allowed us to monitor drug treatment and systemic response to therapy. Moreover, eccrine sweat revealed a potentially predictive metabolic pattern stratifying patients by the extent of the metabolic activity of breast cancer tissue at baseline. Eccrine sweat is derived from the otherwise hardly accessible interstitial fluid and, thus, opens up a new dimension for biomonitoring of breast cancer in secondary and tertiary care. The simple sample collection without the need for trained personnel could also enable decentralised long-term biomonitoring to assess stable disease or disease progression. Eccrine sweat analysis may indeed significantly advance 3PM for the benefit of breast cancer patients.

**Supplementary Information:**

The online version contains supplementary material available at 10.1007/s13167-025-00396-6.

## Introduction

The past decade saw significant efforts to broadly implement predictive, preventive and personalised medicine (3PM) [1–4]. This patient-centred approach, which is also referred to as precision medicine [5, 6], promises predictive diagnostics, targeted prevention and personalised treatments in patient care [5, 7–11]. Therefore, recent years have witnessed a clear trend of tailoring medical practice towards patient subgroups or individual patients [12], and this was paralleled by technological advances of molecular profiling. Several examples exist to guide therapy in clinical practice. The kinase inhibitor imatinib pioneered this approach by targeting the chronic myeloid leukaemia (CML)-specific fusion protein BCR-ABL [13]. Furthermore, genotyping identifies enzyme isoforms that determine a patient’s response to the anticoagulant warfarin and helps finding appropriate dosages [14].

Breast cancer remains the most common malignant tumour in women, with growing incidence over the last 20 years [15]. Breast cancer patients benefit from progress in 3PM by stratification into four different subtypes according to the expression status of the oestrogen receptor, the progesterone receptor and human epidermal growth factor receptor type 2 (HER2) [16], and this guides therapeutic strategies. For example, HER2-positive status is present in about 30% of patients and indicates treatment with a dual HER2-antibody therapy comprised of trastuzumab and pertuzumab [17]. The residual cancer burden (RCB) represents one of the most important prognostic markers in breast cancer [18]. 3PM makes further crucial contributions to breast cancer treatment by identifying new approaches for mechanism-based prognosis [19] or advanced stratification based on underestimated clinical symptoms [20, 21].

**Fig. 1:**
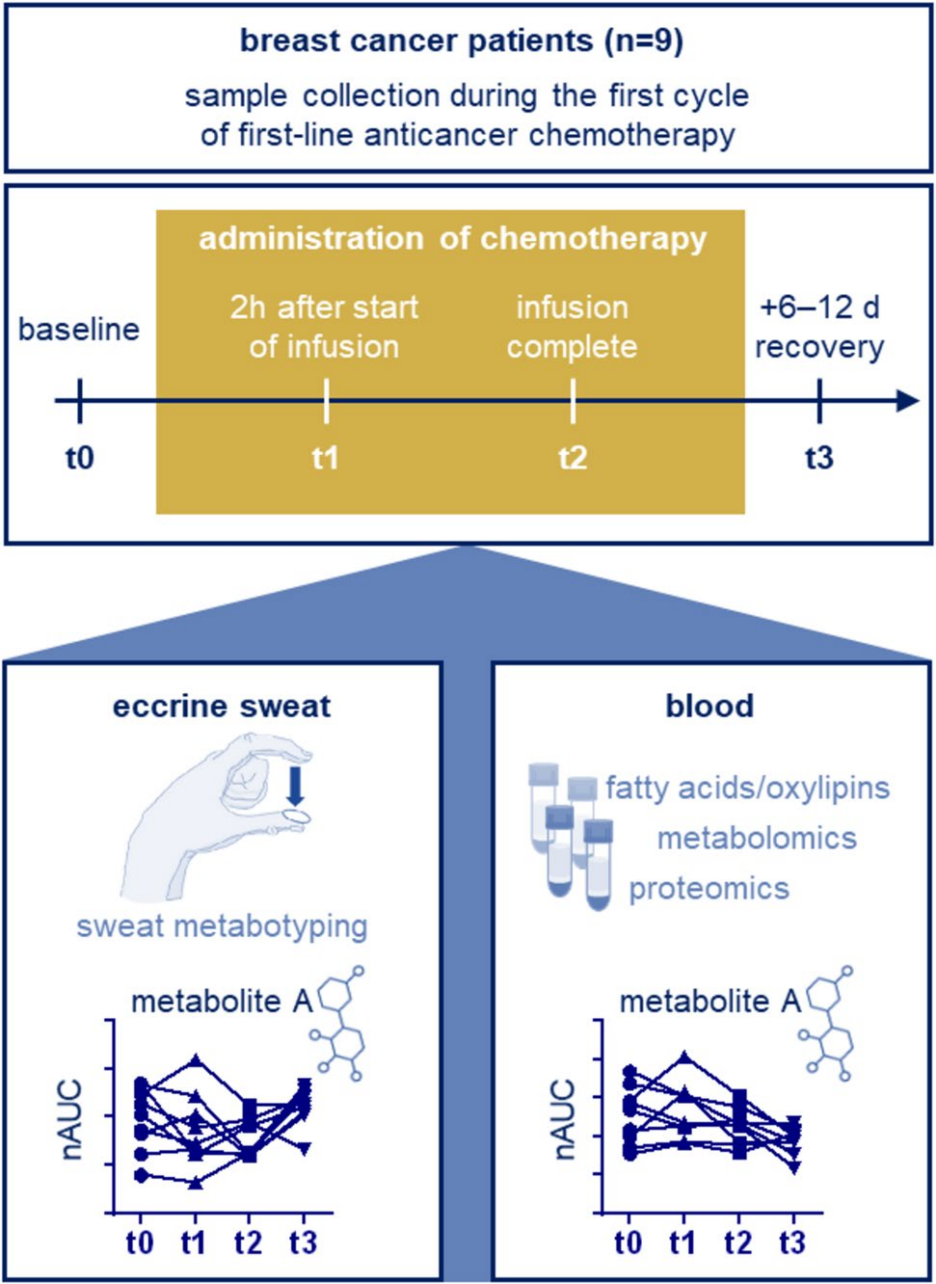
Study design to evaluate eccrine sweat as a relevant biofluid in the context of 3PM in clinical practice. After diagnosis with invasive ductal carcinoma, nine breast cancer patients were monitored during the first cycle of first-line anticancer chemotherapy at four time points by collecting eccrine sweat and blood plasma. In this study, the analysis of eccrine sweat by mass spectrometry enabled drug monitoring, assessing lifestyle parameters, stratification according to metabolite patterns and impact of treatment on disease. Systemic drug effects were comparatively evaluated by sweat metabotyping and plasma multi-omic analysis, the latter integrating metabolomic, proteomic and fatty acid/oxylipin data

Today, 3PM promotes the combination of clinical parameters, as well as environmental and behavioural factors using data-driven bioinformatics [5, 22], with a preference for evidence-based research data. Particularly, molecular phenotyping [23] and large longitudinal studies [5] are expected to significantly contribute to the field. The former also includes post-genomic techniques beyond transcriptomics, e.g. proteomics [24–26] and metabolomics [27, 28], because their integration into clinical multi-omic analysis empowers deep phenotyping of complex disease states and therapeutic interventions [29–31]. The multifactorial and often unclear aetiology of many chronic diseases necessitates post-genomic phenotyping to characterize disease progression, resolution or treatment effects [23, 32–34]. Conducting large longitudinal studies requires innovations in sample collection for molecular phenotyping in the frame of 3PM. We recently reported on an entirely non-invasive biomonitoring technique based on the analysis of eccrine sweat using mass spectrometry [35, 36]. Sweat is underappreciated, but increasingly investigated, as a diagnostic biofluid [37, 38]. The classic approach of sweat analysis includes pilocarpine iontophoresis [39, 40] or wearable microfluidic systems that continuously assess a limited number of parameters [38, 41, 42]. Our mass spectrometry-based approach is capable to discover and profile hundreds of both exogenous molecules and endogenous metabolites in eccrine sweat in short time intervals [35]. Sweat samples are obtained non-invasively from the fingertips by pressing the thumb and index fingers of one hand on a prewetted substrate (Fig. 1) [35, 36], and this approach was analytically validated for several exogenous marker molecules [35]. Interestingly, sweat analysis was suggested to be useful for monitoring certain lifestyle parameters related to environmental pollution, smoking or bioactive small molecules [36]. An alternative approach showed potential for cancer diagnostic purposes based on mass spectrometric peptide analysis of fingerprint smears [43].

Eccrine sweat gland metabolism is commonly responsible for body thermoregulation [44] and in some cases associated with drug excretion [45]. It is also known that some components released in eccrine sweat are derived from the interstitial fluid [44, 46, 47], which may account for the broad range of endogenous end exogenous metabolites found in eccrine sweat [35, 36]. The interstitial fluid is of high medical importance because it represents the site of action of therapeutic agents [48], and access to this compartment is usually challenging. Therefore, eccrine sweat might serve as proxy for interstitial fluid-derived metabolites.

## Working hypothesis in the framework of 3PM

We previously used sweat analysis to assess predetermined lifestyle and metabolic parameters in individuals [35, 36]. Eccrine sweat is an under-investigated biofluid for breast cancer diagnostics. Its collection is entirely non-invasive, risk-free and low cost, and the potential benefit for patients may be significant. This proof-of-concept study reports for the first time on the discovery of metabolic biomarkers from eccrine sweat of breast cancer patients. We use mass spectrometry to analyse a broad range of exogenous and endogenous metabolites in nine breast cancer patients with invasive ductal carcinoma. The patients belonged to the subtypes luminal B, HER2-positive luminal and triple-negative, for whom cytotoxic chemotherapy was indicated. Eccrine sweat and plasma samples were collected at four time-points during the first cycle of neoadjuvant chemotherapy, including baseline. Specifically, eccrine sweat was investigated as a complementary source to the more conventional blood plasma to discover metabolic molecular patterns related to breast cancer treatment and potentially response. Importantly, this study investigates whether the analysis of eccrine sweat can be used to monitor therapy. In addition, evidence-based assessment of lifestyle parameters detected in eccrine sweat that represent risk factors may be objectively included in predictive diagnostics and aid in creating individual treatment algorithms. Finally, this study was designed to evaluate whether eccrine sweat may reveal endogenous metabolic parameters for predictive diagnostics. Thus, eccrine sweat may contribute to secondary and tertiary care 3PM by enabling longitudinal sample collection to access metabolic profiles of patients and inform about disease stabilization or progression. The possibility for decentralised sample collection may promise a future application of eccrine sweat analysis even in primary care in the context of 3PM. Ultimately, analysis of metabolites of eccrine sweat in breast cancer patients may open up a new dimension in medical service in all aspects of 3PM, including predictive diagnostics, targeted prevention and personalisation of medical services.

## Materials and methods

### Cohort description and study design

Eleven female breast cancer patients with invasive ductal carcinoma (gradings 2–3) were initially recruited into this pilot study at the General Hospital Vienna, Austria. All patients gave permission to sampling by signing a written informed consent, and the study was approved by the ethics committee of the Medical University of Vienna (1863/2017). Patient and tumour characteristics were assessed, including age, BMI, histological type and grading (Table 1). The proliferative index according to MIB-1 and the receptor status of all patients was assessed, as well as the RCB class and the occurrence of ductal carcinoma in situ (DCIS). All patients were non-diabetic. Patients P04 and P07 were excluded from the study due to incomplete data points and different type of chemotherapy, respectively.

**Table 1:** Clinical characteristics of the female breast cancer patients included in this pilot study. IDC invasive ductal carcinoma, MIB-1 Ki-67 labelling index using MIB-1 antibody, RCB residual cancer burden, DCIS ductal carcinoma in situ, ER oestrogen receptor, PR progesterone receptor, HER2 human epidermal growth factor receptor 2

Patient N°	Age [yr]	BMI [kg m^−2^]	Histological type	Histological grading	MIB-1 [%]	RCB class	DCIS	Diabetes	Receptor status			Molecular subtype
									ER	PR	HER2	
1	48	25,8	IDC	3	70	I	No	No	3	2	3	HER2 Luminal
2	75	24,0	IDC	2	60	0	Yes	No	0	0	0	Triple-negative
3	58	20,5	IDC	3	40	I	Yes	No	3	0	3	HER2 Luminal
5	67	25,6	IDC	2	30	II	Yes	No	3	2	2	Luminal B
6	42	17,3	IDC	3	50	II	Yes	No	3	3	3	HER2 Luminal
8	31	22,0	IDC	2	30	III	No	No	3	2	1	Luminal B
9	51	25,7	IDC	2	70	I	Yes	No	3	3	2	Luminal B
10	39	23,7	IDC	2	60	II	Yes	No	3	3	0	Luminal B
11	35	24,6	IDC	3	40	II	No	No	0	0	0	Triple-negative

The stratification to specific molecular subtypes determined the cytotoxic chemotherapy treatment schedule. First-line cytotoxic chemotherapy included mainly 5-fluorouracil, epirubicin and cyclophosphamide (FEC). This was individually replaced or complemented by docetaxel, abraxane, carboplatin and/or antibody therapy. The patients also received an anti-emetic drug combination consisting of dexamethasone, ondansetron (zofran) and emend. Except for patient P10, the patients were given G-CSF the day after chemotherapy.

For this study, the patients were monitored during the first cycle of chemotherapy. Samples were obtained at four time points, i.e. (1) before the start of the infusion (baseline, t_0_); (2) 2 h after start of infusion (t_1_); (3) after completion of infusion (t_2_); and (4) 6–12 days after infusion (recovery, t_3_). At these timepoints, matched blood plasma (EDTA) and eccrine sweat from the fingertips were collected for plasma multi-omic analysis and sweat metabotyping, respectively. Individual blood plasma samples were aliquoted for dedicated proteomic, metabolomic as well as fatty acid/oxylipin workflows. Additionally, clinical routine blood parameters were assessed before the first chemotherapy cycle (baseline, t_0_) and 7–10 days after infusion (recovery, t_3_).

### Eccrine sweat collection

Sampling units (precision wipes, Kimtech Science, Kimberly-Clark Professional, USA, 0.5 in. diameter) were prewetted with aqueous solution (3 µL, LC–MS grade H_2_O) and stored in labelled Eppendorf tubes as previously described [35]. Sweat collection involved rinsing hands with warm tap water and subsequent drying with disposable paper towels. After a lag time of 1 min, the sampling unit was placed between thumb and index finger using a clean tweezer and gently held for 1 min. Then, the sampling unit was transferred back to the Eppendorf tube and stored at 4 °C until further processing.

### Eccrine sweat metabolomics

#### Sample processing

Aqueous solution (120 µL, VWR Chemicals, LC–MS grade) containing caffeine-(trimethyl-d9) and N-acetyl-tryptophan-d3 (each 1 pg∙µL^−1^) and formic acid (FA, 0.2%) was added to the sampling unit in the Eppendorf tube. Metabolites were extracted by pipetting up and down 15 times. The sampling unit was pelleted on the bottom of the tube, and the supernatant was transferred into HPLC vials equipped with a 200 µL V-shape glass insert (both Macherey–Nagel GmbH & Co.KG) to be analysed by LC–MS/MS.

#### Data acquisition

A Q Exactive HF (Thermo Fisher Scientific) mass spectrometer was coupled to a Vanquish UHPLC System (Thermo Fisher Scientific). A Kinetex XB-C18 column (100 Å, 2.6 µm, 100 × 2.1 mm, Phenomenex Inc.) was used for chromatographic separation. Mobile phase A consisted of water (0.2% FA) and mobile phase B of methanol (0.2% FA). Water, FA and methanol were purchased from VWR Chemicals (Vienna, AT in LC–MS grade). The following gradient was employed: 1–5% B in 0.3 min and then 5–40% B from 0.3–4.5 min, followed by a column washing phase of 1.4 min at 80% B and a re-equilibration phase of 1.6 min at 1% B resulting in a total runtime of 7.5 min. Flow rate was set to 500 µL min^−1^, the column temperature to 40 °C and the injection volume was 10 µL. All samples were analysed in technical duplicates. Electrospray ionization was performed in positive and negative ionization mode. MS scan range was *m/z* 100–1000, and the resolution was set to 60′000 (at *m/z* 200). The four most abundant ions of the full scan (Top 4) were selected for HCD fragmentation applying 30 eV collision energy. Fragments were analysed at a resolution of 15,000 (at *m/z* 200). Dynamic exclusion was set to 6 s. The instrument was controlled using Xcalibur software (Thermo Fisher Scientific).

#### Data analysis

Intensity extraction was performed as follows: MSConvert and ProteoWizard were used to convert raw files into mzML files. Those were loaded into MZmine (Version 3.4.27) [49] and processed using the modules ADAP chromatogram builder and feature resolver [50]. This was followed by 13C isotope filtering, retention time alignment, blank background subtraction and filtering of features that had invalid isotope patterns. Then, gap filling was performed, duplicates filtered and ion adduct identity determined. Feature information including *m/z* values, retention time (RT), ion identity and areas under the curve (AUCs) in the specific raw files were exported as a.csv file for further processing.

Feature annotation was performed as follows: An.mgf file including precursor and merged MS/MS information was exported from MZmine and loaded into SIRIUS (Version 5.8.0) [51]. The molecular formula identification was performed with the standard orbitrap instrument settings (mass accuracy 5 ppm). The CSI:FingerID module was used to compute MS/MS data into fragmentation trees which were used to determine candidates for possible molecular structures and database searches. The results for the top hits containing a COSMIC score (Confidence score) were exported into a.tsv file to later merge feature IDs, areas and structural information into one single file.

Using RStudio with R (Version 4.3.1), the data exported from MZMine and SIRIUS were loaded and processed to receive a clean data matrix for statistical evaluation. This process included the import of manually integrated internal standard areas (caffeine-(trimethyl-d9) for positive ionization mode and N-acetyl-tryptophan-d3 for negative ionization mode) using Skyline (Version 22.2.0.351) to receive uniform data which was used for the normalization of areas under the curve (nAUCs) accounting for instrument performance. After calculating the means of technical replicates, the intensities were merged with the feature identification data received from SIRIUS. The finished data frame, containing *m/z* values, *RT*, feature ID, annotation and nAUCs, was exported as a.csv file for statistical processing.

Using Perseus (Version 2.0.10.0), the main columns containing the feature nAUC for each sample were first categorically annotated with group information (time points, donors, RCB). All nAUC values were transformed by applying log2 and then adding a fixed value of + 20. Features with less than 50% valid values at every time point were removed. Then, imputation from the lower gaussian distribution was conducted according to standard procedures of down shift 1.8σ and width 0.3σ. Volcano plots and PSA plots were created after filtering for features that had at least 50% COSMIC score according to SIRIUS.

Raw files generated by the Q Exactive HF instrument were manually reviewed using Xcalibur Qual Browser (Version 4.0, Thermo Fisher Scientific).

### Blood plasma collection

Venous blood samples were collected in K3 EDTA tubes and kept at room temperature for exactly 30 min before centrifugation at 4 °C and 2000 g for 15 min. Plasma was aliquoted into five Eppendorf tubes and stored at – 80 °C until further processing.

### Plasma metabolomics

Metabolomics of patient plasma samples (10 µL) were assessed by a targeted assay. We used the MxP® Quant 500 Kit (Biocrates Life Sciences AG, Innsbruck, Austria, product number 21094.12), which enables the detection and (semi)quantification of up to 631 analytes, including acylcarnitines, an alkaloid, an amine oxide, amino acid related metabolites, bile acids, biogenic amines, carboxylic acids, ceramides, cholesteryl esters, cresol, diacylglycerols, dihydroceramides, fatty acids, glycerophospholipids, glycosylceramides, hormones, indole derivatives, nucleobase-related metabolites, sphingolipids, triacylglycerols, the sum of hexoses and one vitamin/cofactor. Of the initial 631 possible metabolites, we excluded those that were found below LOD in more than 50% of samples or that showed an unacceptable accuracy (< 80% or > 120%) based on internal standards or blank measurements. This reduced the number of metabolites to 487. Measurements were carried out using liquid chromatography tandem mass spectrometry (LC–MS/MS) and flow injection (FIA)-MS analyses on a Sciex 6500 + series mass spectrometer coupled to an ExionLC AD chromatography system (SCIEX, Framingham, MA, USA), utilizing the Analyst 1.7.1 software with hotfix 1 (also SCIEX) according to the kit procedure.

The LC–MS method in positive ion mode was performed as follows: The UHPLC autosampler and column oven were held at 10 °C and 50 °C, respectively. The injection volume was 5 µL. Eluent A was water (0.2% formic acid), and eluent B was acetonitrile (0.2% formic acid). A run time of 5.8 min was applied. Eluent B remained at 0% B for 0.25 min, followed by a linear gradient to 12% B at 1.5 min and 17.5% B at 2.7 min. The percentage of eluent B further linearly increased to 50% B at 4 min and 100% B at 4.5 min, where it remained until 5 min. Eluent B was reduced to 0% at 5.1 min and remained at 0% until 5.8 min. The flow rate was 0.8 mL/min until 4.5 min, then increased to 1 mL∙min^−1^ until 4.7 min and remained until 5.1 min. Finally, the flow rate was reduced again to 0.8 mL∙min^−1^ until 5.8 min. A scheduled MRM experiment was run in positive polarity. Detection window was 30 s, target scan time was 0.15 s and 3 ms pause between mass ranges. Q1 and Q3 were held at unit resolution. Source parameters were CUR 45, voltage 5.5 kV, temperature 500 °C, ion source gas at 60 for nebulizer and 70 for Turbo V heater (arbitrary units).

The LC–MS method in negative ion mode was performed as follows: The UHPLC autosampler and column oven were held at 10 °C and 50 °C, respectively. The injection volume was 15 µL. Eluent A was water (0.2% formic acid), and eluent B was acetonitrile (0.2% formic acid). A run time of 5.8 min was applied. Eluent B remained at 0% for 0.25 min, followed by a linear gradient to 25% B at 0.5 min and 50% B at 2.0 min. Eluent B further linearly increased to 75% B at 3 min and 100% B at 3.5 min, where it remained until 5 min. Eluent B was reduced to 0% at 5.1 min and remained at 0% until 5.8 min. The flow rate was 0.8 mL/min until 3.5 min, then increased to 1 mL/min at 4.7 min and remained until 5.1 min. Finally, the flow rate was reduced to 0.8 mL/min until 5.8 min. A scheduled MRM experiment was run in negative polarity. Detection window was 30 s, target scan time was 0.15 s and 3 ms pause between mass ranges. Q1 and Q3 were held at unit resolution. Source parameters were CUR 35, voltage –4.5 kV, temperature 650 °C, ion source gas at 40 for nebulizer and 40 Turbo V heater (arbitrary units).

FIA methods were performed as follows: The UHPLC autosampler was held at 10 °C. eluent was acetonitrile (0.2% formic acid). The injection volume was 20 µL. A run time of 3 min at 100% eluent B was applied. The flow rate was 0.03 mL/min from 0 to 1.6 min and then increased to 0.20 mL/min until 2.4 min and remained until 2.8 min. Then, the flow rate was reduced to 0.03 mL/min until 3 min. The MRM experiments was run in positive polarity with 3 ms pause between mass ranges. Q1 and Q3 were held at unit resolution. Source parameters were CUR 20–30, voltage 5.5 kV, temperature 200 °C, ion source gas at 30–40 for nebulizer, and 50–80 for Turbo V heater (arbitrary units).

The required standards, quality controls (QCs) and eluents were included in the kit, as well as the chromatographic column (product number 21117.1). Phenyl isothiocyanate (Sigma-Aldrich, St. Louis, USA) was purchased separately and was used for derivatization according to the kit manual. Preparation of the measurement worklist as well as data validation and evaluation were performed with the software supplied with the kit (MetIDQ-Oxygen-DB110-3005, Biocrates Life Sciences). Data was normalized according to median QC level 2.

### Plasma oxylipin and fatty acid analysis

#### Sample preparation

Oxylipins and fatty acids were enriched from blood plasma similarly to a previous report [30]. Briefly, plasma (400 µL) was freshly thawed on ice and proteins were precipitated with cold ethanol (1.6 mL, abs. 99%, –20 °C; AustrAlco). Ethanol contained an internal standard mixture of 12S‐HETE‐d8, 15S‐HETE‐d8, 5‐Oxo‐ETE‐d7, 11,12‐DiHETrE‐d11, PGE2‐d4 and 20‐HETE‐d6 (each at 100 nm, Cayman Europe, Tallinn, Estonia, Supplementary Table S1). After centrifugation (30 min, 4536 g, 4 °C), supernatants were transferred into new Falcon™ tubes (15 mL), and ethanol was evaporated via vacuum centrifugation at 37 °C until the original sample volume (400 µL) was restored. Solid phase extraction was performed by loading samples with Pasteur pipettes onto pre-conditioned StrataX SPE columns (30 mg∙mL^−1^, Phenomenex, Torrance, CA, USA). After washing with water (5 mL, LC–MS grade, VWR) samples were eluted with ice-cold methanol (500 µL, VWR) containing 2% FA. Methanol was evaporated under a gentle stream of dinitrogen at room temperature. The dried samples were then reconstituted in 150 µL reconstitution buffer (v/v 65: 31.5: 3.5 water: acetonitrile: methanol + 0.2% FA) for data acquisition.

#### Data acquisition

Chromatographic separation was performed using a Thermo Scientific™ Vanquish™ (UHPLC) system equipped with a Kinetex® C18‐column (2.6 µm XB-C18 100 Å, 150 × 2.1 mm, Phenomenex Inc.). The mobile phase A consisted of water + 0.2% FA and mobile phase B consisted of acetonitrile/methanol (v/v 90:10) + 0.2% FA. A gradient flow profile was applied starting at 35% B and increasing to 90% B (1–10 min), further increasing to 99% B within 0.5 min and held for 5 min. Solvent B was then decreased to the initial level of 35% within 0.5 min, and the column was equilibrated for 4 min. The total run time was 20 min per sample. The flow rate was kept at 200 μL min^−1^ and the column oven temperature at 40 °C. The injection volume was 20 µL, and all samples were analysed in technical duplicates. Mass spectrometric analysis was accomplished with a Q Exactive™ HF orbitrap (Thermo Fisher Scientific, Austria), equipped with a HESI source in negative, as well as positive ionization mode. A spray voltage of ± 3.5 kV and a capillary temperature of 253 °C were applied. Auxiliary gas was set to 10 a.u. and sheath gas was set to 46 a.u. The MS scan range was set to *m/z *250–700 with a resolution of 60′000 (at *m/z* 200) on the MS1 level. A Top 2 method was selected, as well as an HCD fragmentation with normalized collision energy of 24. An inclusion list covering 33 m*/z*-values specific for well-known eicosanoids and MS precursor molecules was imported (Supplementary Table S2). The resulting fragments were additionally analysed on the MS2 level at a resolution of 15′000 (at *m/z* 200).

#### Data analysis

For data analysis, analytes were compared with an in-house established database on the MS1 level based on exact mass and retention time (degree of identification shown in Supplementary Table S3) using the TraceFinder software (version 4.1). Subsequently, MS/MS fragmentation spectra were manually compared with reference spectra of in-house measured, commercially available standards or to reference spectra from the Lipid Maps depository library (July 2018). Relative quantification of the identified analytes was then performed on the MS1 level using the TraceFinder software (version 4.1). The resulting peak areas were loaded into an R software package environment (Version 4.2.0) and log2-transformed. The mean peak area of the internal standards was subtracted from the analyte peak areas to correct for variances arising from sample extraction and LC–MS/MS analysis. Then, each log2-transformed area was increased by adding (x + 20) enabling missing values imputation. Missing values were imputed using the minProb function of the imputeLCMD package (Version 2.1).

### Plasma proteomics

#### Sample processing

Plasma samples (EDTA-anticoagulant) were diluted 1:20 in lysis buffer (8 m urea, 50 mm triethylammonium bicarbonate (TEAB), 5% sodium dodecyl sulphate (SDS)) and heat denatured (95 °C, 5 min). The protein concentration was determined using a bicinchoninic acid (BCA) assay. A total of 20 µg protein per sample was digested using the ProtiFi S-trap approach. Solubilised protein is reduced with dithiothreitol (64 mm) and thiols carbamidomethylated using iodoacetamide (48 mm). After diluting with trapping buffer (90% v/v methanol, 0.1 M TEAB), the samples were loaded onto S-trap mini cartridges. Samples were extensively washed and then digested with trypsin/Lys-C (1:40) at 37 °C (2 h). Peptides were eluted, dried and stored at –20 °C.

#### Data acquisition

Plasma proteomic analysis was performed as described previously [30]. Dried peptide samples were dissolved with 5 µL FA (30%) containing synthetic peptide standards and additionally with 40 µL loading solvent (97.95% water, 2% acetonitrile, 0.05% trifluoroacetic acid) and transferred to HPLC vials equipped with a 200 µL V-shape glass insert (both Macherey–Nagel GmbH & Co.KG). A Dionex UltiMate3000 nanoLC-system (Thermo Fisher Scientific) was used for chromatographic separation. The injection volume was 1 µL, and the peptides were pre-concentrated on a pre-column (C18 Pepmap100, 2 cm × 75 µm, Thermo Fisher Scientific) at a flow rate of 10 µL∙min^−1^ using mobile phase A (99.9% H2O, 0.1% FA). Subsequent peptide separation was achieved on an analytical column (1.6 µm C18 Aurora Series emitter column, 25 cm × 75 µm, from IonOpticks) by applying a flow rate of 300 nL min^−1^ and using a gradient of 7–40% mobile phase B (79.9% acetonitrile, 20% water, 0.1% FA) in 43 min. The total LC run was 85 min including washing and equilibration steps. Mass spectra of peptides were acquired in a data-dependent analysis mode using a timsTOF Pro mass spectrometer (Bruker Daltonics) equipped with a captive spray ion source (1650 V). The instrument was operated in the Parallel Accumulation-Serial Fragmentation (PASEF) mode, and a moderate MS data reduction was applied. The scan range was set to *m/z* 100–1700 and 0.60–1.60 V s cm^−2^ with a ramp time of 100 ms. All experiments were performed with 10 PASEF MS/MS scans per cycle leading to a total cycle time of 1.16 s. The collision energy was ramped as a function of increasing ion mobility from 20 to 59 eV and the quadrupole isolation width were set to 2 Th for *m/z* < 700 and 3 Th for *m/z* > 700.

#### Data analysis

Protein abundance profiles were obtained by label-free quantification (LFQ) of the proteomics data. Using the publicly available software package MaxQuant (Version 1.6.17.0) together with the Andromeda search engine, raw data was searched against the SwissProt *homo sapiens* database (Version 141,219 containing 20′380 entries) including an allowed peptide tolerance of 20 ppm, a maximum of two missed cleavages, carbamidomethylation on cysteines as fixed modification as well as methionine oxidation and N-terminal protein acetylation as variable modifications. A minimum of one unique peptide per protein was set as search criterium for positive identifications. Additionally, the “match between runs” option was applied, using a 0.7 min match time window and a match ion mobility window of 0.05 as well as a 20 min alignment time window and an alignment ion mobility of 1. A false discovery rate (FDR) of ≤ 0.01 was set for all peptide and protein identifications. The identified proteins were filtered for reversed sequences as well as common contaminants and annotated according to the different study groups using Perseus software (Version 1.6.14.0). Then, LFQ intensity values were log2-transformed, and proteins were additionally filtered for their number of independent identifications. Protein had to be conditionally identified in 70% of at least one sample group. Finally, missing values were replaced from a normal distribution with width 0.3σ and down shift 1.8σ.

### Statistical analysis

#### Eccrine metabolomics

The untargeted metabolomics from eccrine sweat included a COSMIC confidence score of ≥ 50% for feature identification [52]. One-way ANOVA statistics were applied to evaluate significant metabolite time courses with four time points and nine subjects per time point. The volcano plots to compare the eccrine sweat metabolome of specific time points (t_0_ vs t_1_) or of RCB comparisons (at t_0_ or t_3_) included two-sided t-tests. These *P* values were multiple testing corrected based on false discovery rate of 0.05, including 250 permutations and were calculated in Perseus software (Version 2.0.10.0).

### Plasma metabolomics

One-way ANOVA statistics were applied to evaluate significant metabolite time-courses with four time points and nine subjects per time point. For plasma metabolomics, the volcano plots of RCB comparisons (at t_0_ or t_3_) were obtained by plotting Log2-fold changes and permutation-based (*n* = 250) multiple testing corrected P-values. A Log2-fold change cut-off of ± 1 and a –Log10 (adjusted *P* value) of 1.3 was chosen.

#### Plasma fatty acids/oxylipins

One-way ANOVA statistics were applied to evaluate significant metabolite time-courses with four time points and nine subjects per time point. RCB comparisons (at t_0_ or t_3_) of single fatty acids/oxylipins were analysed for significant changes between groups using Kolmogorov–Smirnov tests.

#### Plasma proteomics

A false discovery rate of 0.01 for identification was applied on protein and peptide levels. One-way ANOVA statistics were applied to evaluate significant metabolite time-courses with four time points and nine subjects per time point. Comparisons of time points were calculated by paired t-tests using multiple testing corrected *P* values, based on false discovery rate of 0.05, including 250 permutations. Those were performed in Perseus software (Version 2.0.10.0).

#### Clinical parameters

Single parameters were evaluated for significant changes by a paired t-test between baseline (Pre) and recovery (Post).

## Results

### Study description

Eleven female breast cancer patients diagnosed with invasive ductal carcinoma were recruited for this patient-centred proof-of-concept study at the General Hospital Vienna, Austria. Patients P04 and P07 were excluded from analysis due to incomplete data points and different type of chemotherapy, respectively. The remaining nine breast cancer patients (*n* = 9) displayed an age distribution of 50 ± 15 yr and a BMI of 23.2 ± 2.9 kg∙m^−2^. All patients were non-diabetic. Individual tumour characteristics were histologically assessed, and receptor status was evaluated (Table 1). The patients presented molecular tumour subtypes corresponding to luminal B, HER2-positive luminal and triple-negative breast cancer. Patients of the luminal A subtype were not included, as chemotherapy has shown a poor effect in this subtype [37, 53, 54]. The proliferative activity was 30–70% [55]. According to the receptor status, the patients received individually tailored anticancer chemotherapy that typically relied on the combination of epirubicin + cyclophosphamide, except for patients P01 and P10, who received docetaxel and abraxane alone, respectively. HER2-positive patients received trastuzumab and pertuzumab additionally to their respective cytotoxic agents. Concomitantly, anti-emetic medication was administered to all patients consisting of dexamethasone, ondansetron and aprepitant (see Supplementary Data 1 for treatment details).

Eccrine sweat and plasma samples were collected at four time points over the course of the first cycle of chemotherapy (Fig. 1), corresponding to (1) before drug administration (baseline, t_0_), (2) 2 h after starting the infusion (t_1_), (3) directly after infusion completion (t_2_) and (4) 6–12 days after infusion (recovery, t_3_). The method and timing of blood collection and plasma preparation have significant impact on sample analysis and evaluation. Therefore, a precise protocol for sample management based on a community-initiated position paper was implemented to ensure reproducible sampling [56]. Eccrine sweat samples were analysed by liquid chromatography-tandem mass spectrometry (LC–MS/MS). Blood plasma was aliquoted for subsequent plasma multi-omic analysis, including metabolomics, fatty acids/oxylipins, as well as proteomics, to ensure one freeze–thaw cycle. Each omics-platform was performed according to a dedicated sample preparation and LC–MS/MS workflow [29, 30]. Thus, a total of 144 omics analyses were carried out for this study. A separate aliquot of blood plasma was used to assess routine clinical parameters at baseline (t_0_) and recovery (t_3_). Eccrine sweat collection was well accepted among patients. In this study, plasma multi-omics was employed to obtain an overview about the systemic impact of first-line cytotoxic therapy in these breast cancer patients, which was used as a framework in the interpretation of the interstitium-derived eccrine sweat.

### The metabolic composition of eccrine sweat is a proxy for the interstitial fluid

Eccrine sweat was collected as previously described [35]. Thumb and index finger were pressed on a prewetted sampling unit (approximately 1.27 cm^2^) for 1 min, and the resulting sample was processed by extracting metabolites under aqueous conditions. Mass spectrometry-based analysis of eccrine sweat revealed 1561 small molecule features from combined positive and negative ionization modes of the 36 samples. Of these, 247 (16%) distinct metabolites were successfully annotated by accurate mass, fragmentation spectrum and retention time and showed a COSMIC confidence score of ≥ 50% [52].

**Fig. 2:**
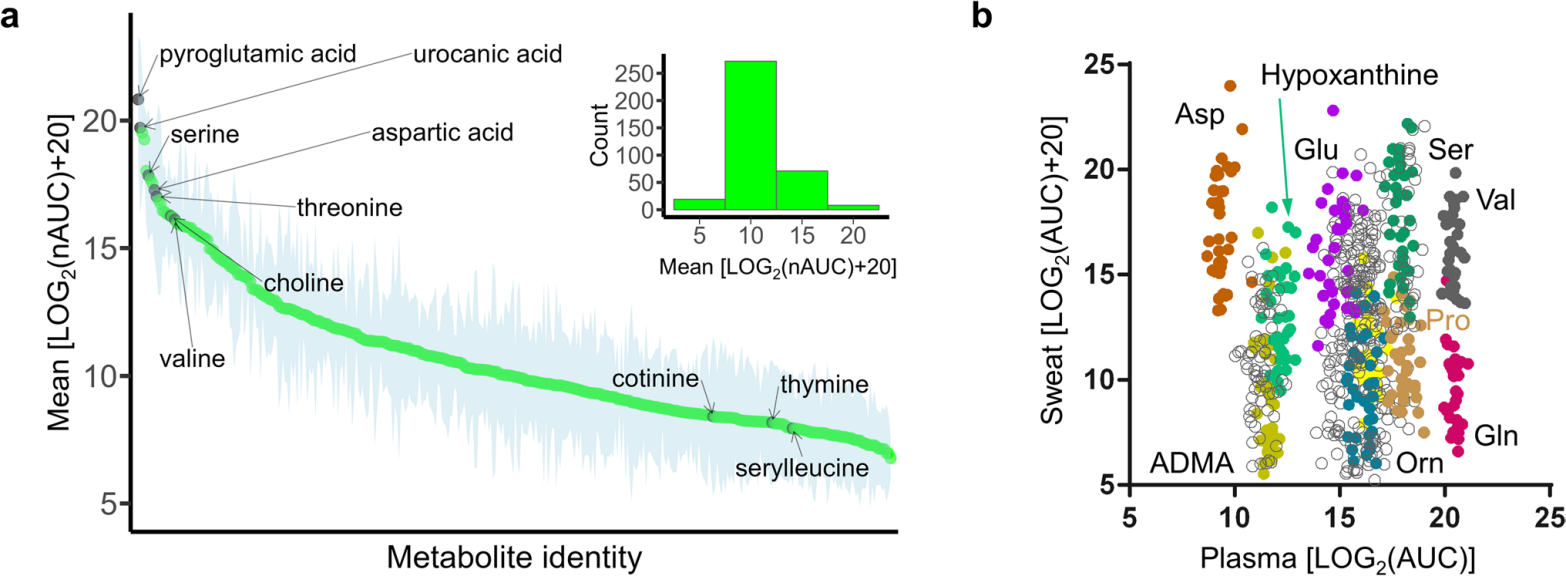
Eccrine sweat features a specific metabolic profile. **a** Ranked median and standard deviation of LOG2-transformed and normalized area under the curve (nAUC) values (*n* = 36) of the 247 annotated metabolites in eccrine sweat of breast cancer patients. The inset shows the frequency distribution of metabolite abundance. **b** Scatter plot showing the direct comparison of metabolite abundance in eccrine sweat and plasma samples as log2-transformed AUC and area under the curve (AUC) values. The scatter plot includes 21 metabolites from all 36 samples resulting in 756 data points

Eccrine sweat is supposed to originate from the interstitial fluid, and its metabolic composition is believed to be largely determined by this compartment [38, 44]. The metabolic profile confirmed that eccrine sweat has a specific composition that is distinct from blood plasma (Fig. 2a) [57]. Median normalized intensity levels of successfully annotated metabolites were detected over a dynamic range of four orders of magnitude. A small number of metabolites seemed particularly abundant, including pyroglutamic acid, urocanic acid, serine, aspartate, threonine, choline and valine. At the lowest intensity, we identified thymine, serylleucine and cotinine.

Plasma metabolites were analysed using the validated Biocrates MxP® Quant 500 kit based on targeted mass spectrometry, and 487 metabolites were identified. Due to the different sample preparation acquisition strategies of eccrine sweat and plasma analysis, 21 metabolites were identified in both approaches that correspond to 16 amino acids(-related), 2 carnitines and 2 nucleobases related molecules and choline. For these metabolites, a scatter plot was created by using log2-transformed areas under the curve (AUCs) of matched sweat and plasma samples giving 756 data points and revealed non-random patterns (Fig. 2b). This transformation allowed metabolites to populate approximately the same intensity range. Each metabolite clustered in specific regions of the scatter plot suggesting an active regulation of these metabolites in the respective compartments. Moreover, while aspartate (Asp) was only detected below limits of detection in plasma, this metabolite was among the most intense representatives in eccrine sweat. Conversely, glutamine (Gln) was highly abundant in plasma, but showed low intensity in eccrine sweat.

The variation of sweat-derived metabolites was higher compared with the same metabolites in plasma (Fig. 2b). Overall, the d9-caffeine-normalized AUC (nAUC) intensity values of sweat-derived metabolites featured a median CV of 156%. Their CV distribution did not correlate significantly with intensity, nor retention time (Supplementary Fig. S1). The higher CVs of sweat-derived metabolites most probably stem from differences in sweat rates. The qualitative and quantitative analysis of metabolites from eccrine sweat is representative of the interstitial space and allows to access the systemic consequence of breast cancer in this compartment.

### Eccrine sweat enables drug monitoring in breast cancer patients

**Fig. 3:**
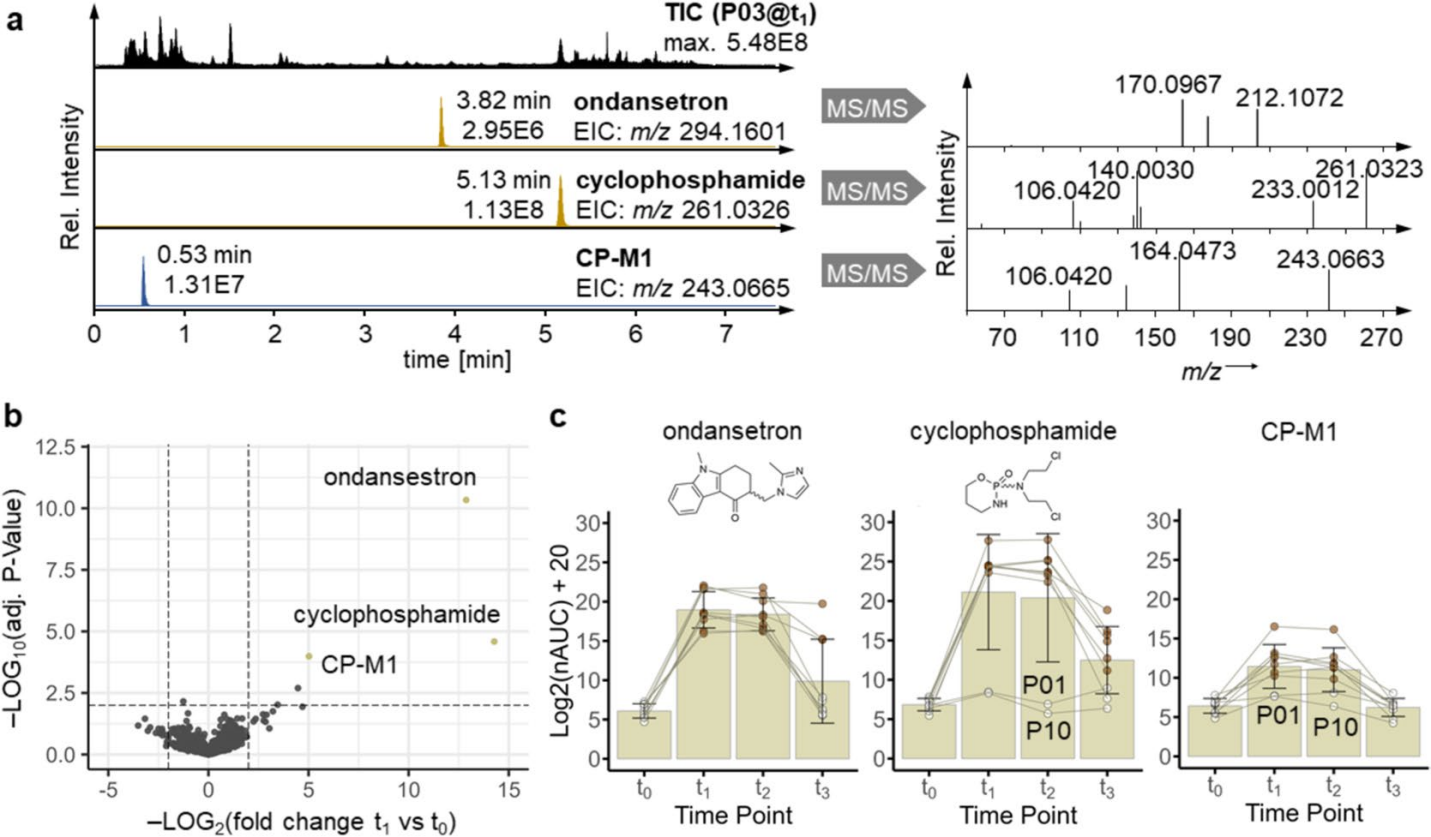
Eccrine sweat can be used for drug monitoring in breast cancer patients. **a** The total ion chromatogram (TIC) of a mass spectrometry-based analysis of metabolites from eccrine sweat is shown of patient P03 during administration at t_1_. Extracted ion chromatograms (EICs) of small molecule drugs detected in the eccrine sweat of the same patient are also shown, including retention time, intensity and associated tandem mass spectra (MS/MS). **b** The volcano plot contrasts the metabolic profile of eccrine sweat from all nine patients during the administration phase (t_1_) compared with baseline (t_0_). Ondansetron, cyclophosphamide and an associated impurity of cyclophosphamide (CP-M1) are the most significantly upregulated molecules. **c** The bar charts show individual time-dependent abundance changes of ondansetron, cyclophosphamide and CP-M1 in eccrine sweat over the first cycle of chemotherapy. Metabolites not detected in a given sample are shown as white circles

As the composition of eccrine sweat is determined by the interstitial fluid and benefits from simple sampling, it may represent a suitable matrix for drug monitoring during treatment cycles. An exemplary total ion chromatogram (TIC) of eccrine sweat is shown of patient P03 during the administration phase at t_1_ (Fig. 3a). Next to endogenous metabolites, small molecule components of the treatment were detected in eccrine sweat. Comparing the metabolomic profiles of all nine patients at baseline t_0_ to the one after the start of the administration at t_1_ revealed cyclophosphamide (Log2(FC) = 14.3, –Log(adj. *P* value) = 4.6) and ondansetron (Log2(FC) = 12.9, –Log(adj. *P* value) = 10.3) as the most strongly upregulated molecules (Fig. 3b). Cyclophosphamide and ondansetron are representative of the anticancer chemotherapy and anti-emetic drug combination, respectively, and were verified by external standards. Importantly, despite the relatively high overall CVs of the sweat-derived metabolites, the magnitude of these fold-changes far exceeded their respective CVs and resulted in significant regulatory events. A known degradation product of cyclophosphamide was also detected, i.e. oxadiazaphosphacyclononane (CP-M1, RT = 0.53 min). This compound was observed in all samples, where cyclophosphamide was detected, but at approximately 1000-fold lower intensity, highlighting the dynamic changes of the applied methodology (Fig. 3c, Supplementary Fig. S2).

In addition to the detection of therapeutic agents at individual time points, time-dependent intensity changes were also detected. This demonstrates the possibility of obtaining dynamic marker signatures from eccrine sweat. Ondansetron displayed increased intensity during the administration phase at t_1_ and t_2_ in all nine patients compared to baseline and those returned largely to baseline intensity at recovery (Fig. 3c). All patients received ondansetron according to the treatment schedule. Cyclophosphamide featured a highly similar time-dependent abundance pattern in eccrine sweat (Fig. 3c). However, the increase at t_1_ compared with baseline (t_0_) was not observed in all patients, which also explains the lower *P* value compared with ondansetron (Fig. 3b). Indeed, P01 and P10 did not receive a FEC-based therapy according to the treatment schedule. Generally, ondansetron and cyclophosphamide were not detected at baseline (Fig. 3c, white circles), which is also illustrated in a profile plot over the entire data set (Supplementary Fig. S2). It may be noted that FEC-based therapy was given intravenously, but cyclophosphamide was detected in all cases as one of the most abundant small molecules in eccrine sweat. Eccrine sweat enables both qualitative and quantitative drug monitoring and may be useful for the design of individual treatment plans and therefore, aid in the personalisation of medical services. Additionally, elevated abundances of ondansetron and cyclophosphamide at the recovery time point suggested a lower clearance of these compounds in the respective patients.

### Eccrine sweat enables monitoring of systemic effects of disease and therapy in breast cancer patients

The treatment schedule included both anticancer and anti-emetic therapy and systemic effects may potentially be caused by either medication. For each timepoint, we collected matched eccrine sweat and plasma samples. Therapy-related systemic effects were assessed by evaluating significant differences of single metabolic parameters in the nine breast cancer patients over the treatment cycle using ANOVA-statistics.

#### Sytemic Effects of Therapy in Eccrine sweat

**Fig. 4:**
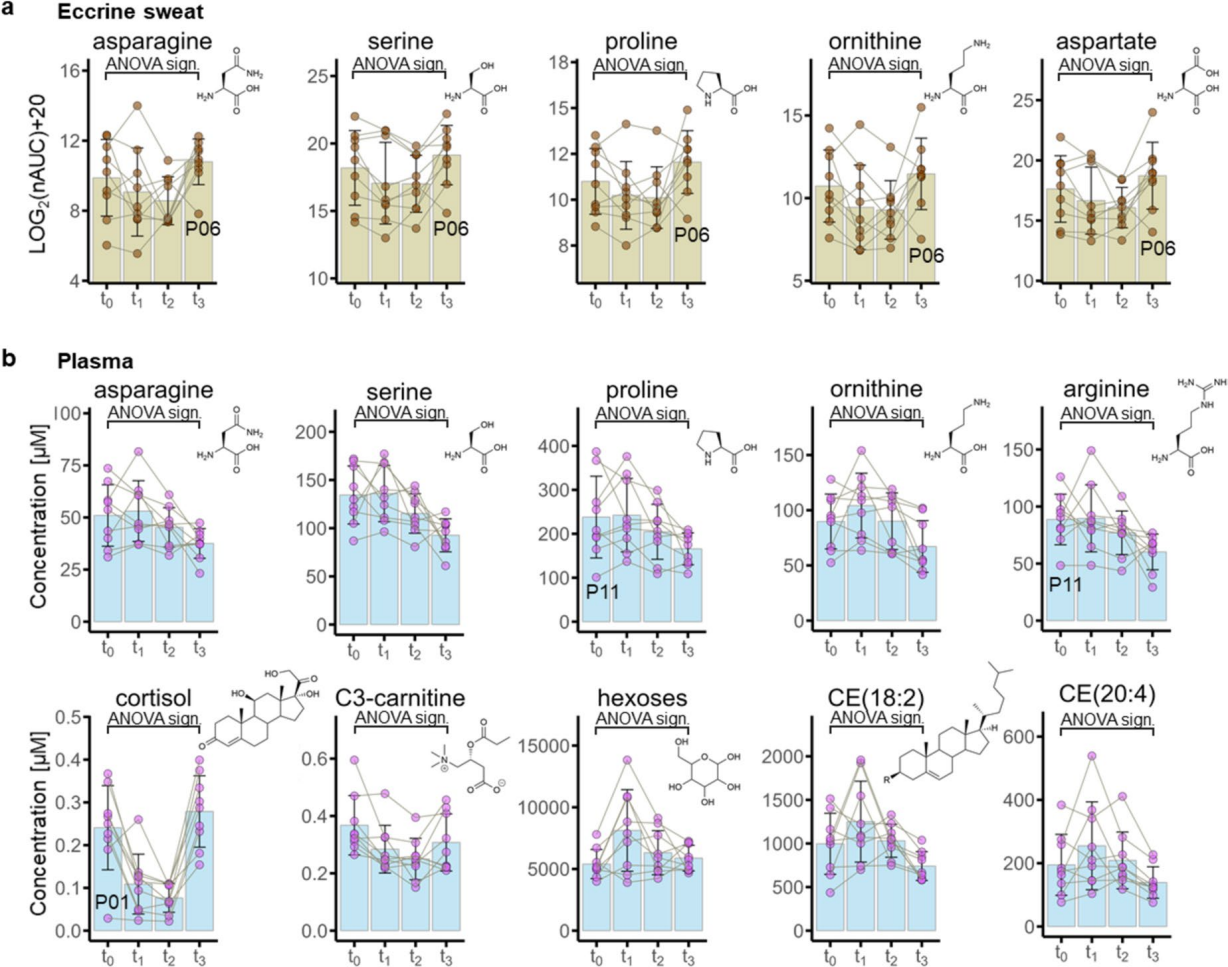
Effect of therapy on eccrine sweat and plasma metabolites in breast cancer patients. ANOVA-testing revealed therapy-related and ANOVA-significantly regulated metabolites in eccrine sweat **a** and plasma **b**. Non-essential amino acids and an amino acid-related metabolite were significantly regulated in both compartments. Plasma metabolites additionally included significantly regulated cortisol, carnitines, hexoses and cholesteryl esters. Other metabolite classes were not found significantly regulated

Eccrine sweat revealed only few endogenous metabolites that potentially reflect systemic responses to therapy. Of the 247 annotated species, 5 amino acids and 1 amino acid-related metabolite featured an ANOVA-significant regulation over time. These included asparagine (*P* value = 0.050), serine (*P* value = 0.021), proline (*P* value = 0.034), ornithine (*P* value = 0.026), aspartate (*P* value = 0.011, Fig. 4a) and prolinamide (*P* value = 0.018, Supplementary Fig. S3a). They all displayed a similar time-dependent progression and an increased intensity at recovery (t_3_) compared with the administration phase (t_2_), except for P06. Other metabolite classes were not significantly regulated.

#### Sytemic Effects of Therapy in Blood plasma

Blood plasma was investigated by a plasma multi-omic analysis, including metabolomics, fatty acids/oxylipins as well as proteomics. Routine clinical parameters were also accessed at baseline (t_0_) and recovery (t_3_). Routine clinical blood parameters of the patients were largely in norm at baseline (Supplementary Data 2).

#### Metabolomics

The Biocrates MxP® Quant 500 kit follows a targeted metabolomics approach. Sample preparation included derivatization with phenyl isothiocyanate and methanolic extraction. The processed samples were analysed by LC–MS and flow-injection analysis tandem mass spectrometry (FIA-MS/MS). By using ANOVA statistics, 20 metabolites were found to be significantly regulated, mainly corresponding to amino acids, amino acid-related metabolites, cortisol and cholesteryl esters. The by ANOVA testing significantly regulated amino acids and amino acid-related metabolites corresponded to asparagine (*P* value = 0.0085), serine (*P* value = 0.0036), proline (*P* value = 0.0159), ornithine (*P* value = 0.0036) and arginine (*P* value = 0.0254, Fig. 4b), as well as methionine (*P* value = 0.0250) and citrulline (*P* value = 0.0078, Supplementary Fig. S3b). Although the concentration ranges of these metabolites were all in norm, they featured a consistent decrease over time in all nine patients.

Cortisol (*P* value = < 0.0001) levels were found to be considerably reduced during the administration phase at t_1_ and t_2_, compared with baseline, but recovered again to normal levels at t_3_. The cortisol levels at t_1_ and t_2_ were even found to be below normal reference values of cortisol in the blood (Fig. 4b). Similarly, propionyl-carnitine (C3-carnitine, *P* value = 0.0057) showed also reduced plasma levels during the administration phase. In contrast, the hexose levels (*P* value = 0.049) increased in most patients during the administration phase, except for P05 and P09, and even transiently exceeded normal reference values for blood sugar. Hexose levels largely normalized again at recovery (t_3_). Finally, the cholesteryl esters CE18:2 (*P* value = 0.030) and CE 20:4 (*P* value = 0.038) were also significantly down-regulated during at recovery. Clinical parameters revealed that cholesterol concentrations (*P* value = 0.0003) also decreased significantly over the therapy cycle (Supplementary Fig. S3c).

#### Fatty acids and oxylipins

A total of 17 fatty acids, 56 oxylipins and 7 bile acids, including isoforms, were detected in blood plasma of these patients using a data-dependent LC–MS acquisition mode after enrichment by solid phase extraction. Of these, we found three lipid species to be significantly regulated, including 9-hydroxyoctadecadienoic acid (9-HpODE, *P* value = 0.0079) and 13-oxo-octadecadienoic acid (13-Oxo-ODE, *P* value = 0.0035) (Supplementary Fig. S3c). The two oxylipins are lipoxygenase products of linoleic acid.

#### Proteomics

Proteomics data was acquired in a data-dependent acquisition mode using a label-free quantification strategy. A total of 425 protein groups were identified in plasma of which 321 were found in at least three out of four samples and in at least one patient. As might be expected from slow onset cytotoxic chemotherapy, we did not find significant protein regulations when comparing different time points using ANOVA-testing. However, we found haemoglobin subunit delta (HBD, *P* value = 0.026) and gelsolin (*P* value = 0.0009) to be significantly down-regulated in the nine patients upon administration of cytotoxic chemotherapy compared with baseline (Supplementary Fig. S3d). This was paralleled by lower numbers of erythrocytes in the clinical parameters, while the total protein amount remained constant (Supplementary Fig. S3d). Gelsolin is an actin-scavenger [58] and its downregulation was accompanied by an upregulation of cytoplasmic actin 1 (ACTB) at the same timepoint (*P* value = 0.013).

### Eccrine sweat reveals metabolic parameters for predictive diagnostics in breast cancer patients

After completion of the respective chemotherapy regimen and surgical removal of the tumour mass, the RCB of the breast cancer patients was determined. The patient cohort was stratified into two groups according to RCB low (class 0–I, *n* = 4) and RCB high (class II–III, *n* = 5) and associated metabolic patterns were evaluated at baseline (t_0_) and at recovery (t_3_) in eccrine sweat.

#### Eccrine sweat for predictive diagnostics

**Fig. 5:**
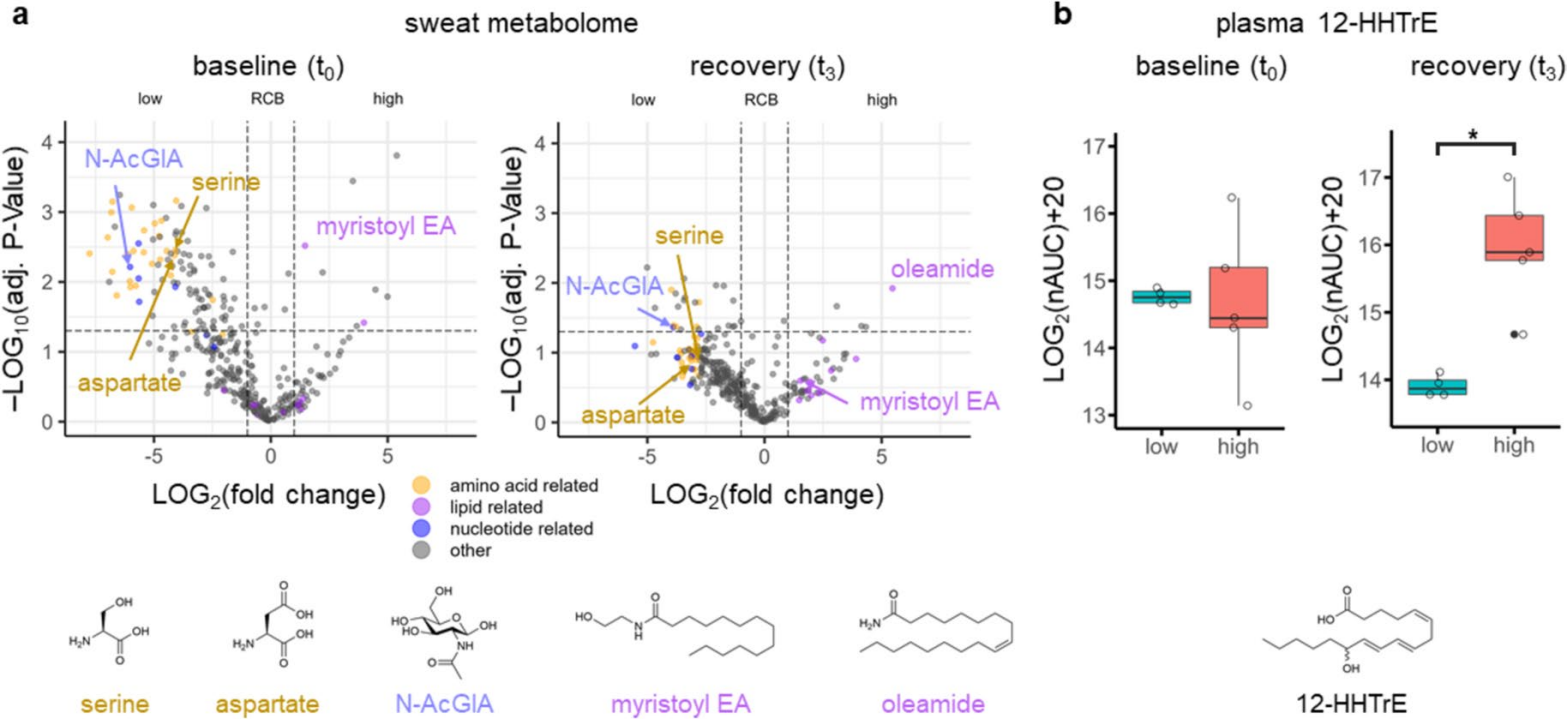
Comparison of RCB low (class 0–I, *n* = 4) and high (class II–III, *n* = 5) groups revealed distinct metabolic patterns at baseline (t_0_) and recovery (t_3_). **a** Volcano plots of the metabolome of eccrine sweat at the indicated time points. The colour code indicates amino acid(-related) (yellow), lipid related (purple) and nucleotide-related (blue) metabolites, including N-AcGlA = N-acetylglucosamine. **b** 12-HHTrE represents the sole significantly regulated oxylipin according to RCB (*P* value = 0.016) at recovery (t_3_). Proteins were not found regulated according to RCB

Patient stratification at baseline (t_0_) generated significant metabolite patterns associated with RCB low in the top left quadrant and RCB high in the top right quadrant in eccrine sweat (Fig. 5a). The RCB low group featured increased abundances of metabolites related to amino acids, e.g. several dipeptides, two cyclic dipeptides, three N-acetylated amino acids and several classic amino acids (yellow circles). The latter included the essential amino acids phenylalanine (Log2(FC) = –6.14, –Log(adj. *P* value) = 2.4), threonine (Log2(FC) = –5.42, –Log(adj. *P* value) = 2.74), valine (Log2(FC) = –2.76, –Log(adj. *P* value) = 30.6), histidine (Log2(FC) = –6.78, –Log(adj. *P* value) = 2.14) and tryptophan (Log2(FC) = –5.88, –Log(adj. *P* value) = 2.44), the non-essential amino acids serine (Log2(FC) = –3.52, –Log(adj. *P* value) = 2.32), aspartate (Log2(FC) = –4.46, –Log(adj. *P* value) = 2.44) and additionally, urocanic acid (Log2(FC) = –2.69, –Log(adj. *P* value) = 1.65). Nucleotide-related metabolites were also observed in this group, including uracil (Log2(FC) = –3.45, –Log(adj. *P* value) = 1.82) and beta-uridine (Log2(FC) = –4.08, –Log(adj. *P* value) = 1.93, blue circles), as well as N-acetyl-glucosamine (Log2(FC) = –6.02, –Log(adj. *P* value) = 2.21). In contrast, the RCB high group displayed a smaller number of regulated lipid-related metabolites, including myristoyl ethanolamine (Log2(FC) = 1.46, –Log(adj. *P* value) = 2.52, purple circles). An additional direct visual comparison of the metabolic differences according to the two RCB groups at baseline in eccrine sweat is provided by the extracted ion chromatograms of individual patients (Supplementary Fig. S4). The same trend of RCB grouping was observed at recovery (t_3_), but a smaller number of metabolites passed the significance threshold (*P* value < 0.05), including urocanic acid (Log2(FC) = –3.89, –Log(adj. *P* value) = 1.39), N-acetyl-glucosamine (Log2(FC) = –3.89, –Log(adj. *P* value) = 1.36) and oleamide (Log2(FC) = 5.46, –Log(adj. *P* value) = 1.92).

#### Blood plasma for predicitve diagnostics

Stratification according to RCB low and high did not reveal regulated proteins, fatty acids or oxylipins at baseline, but 12-hydroxy-heptadecatrienoic acid (12-HHTrE, (Log2(FC) = 2.05, –Log(*P* value) = 2.60) was found as the sole significantly regulated oxylipin at recovery (t_3_, Fig. 5b). Finally, metabolites corresponding to individual lifestyle parameters were also identified, which is a notable point for evidence-based risk factor assessment. For example, nicotine and cotinine were simultaneously detected at considerable intensity in patient P01, who was a declared smoker (Supplementary Fig. S5a). The identification of the metabolite cotinine together with nicotine allows to discriminate between smokers and non-smokers. Regular smokers also revealed other metabolites such as 3-hydroxycotinine in eccrine sweat [36]. A surface contamination becomes increasingly improbable by the observation of metabolic products. Metabolites of the local sampling environment were also observed, corresponding to hexetidine (glypesin) and dehydroacetic acid (biocide 470F). Those are antibacterial agents used in hospitals and were detected in virtually all patients at different intensity (Supplementary Fig. S5b). Hexetidine is the active ingredient in the widely used skin disinfectant “Isozid-H” in hospitals in Austria. The ease of eccrine sweat collection and assessment of metabolic parameters may therefore improve the risk factor assessments in the frame of 3PM and complement questionnaires by more objective evidence-based data.

## Discussion

Eccrine sweat from the fingertips enables assessments of metabolic dynamics [35], as well as lifestyle-derived metabolites [36]. In contrast to sensor platforms [37, 38, 43], mass spectrometry-based analysis allows the detection of hundreds of metabolites and enables the generation of comprehensive individualised metabolic profiles from eccrine sweat. The qualitative and quantitative determination analysis of metabolites from eccrine sweat promises to advance 3PM policies. Here, the potential of metabolite discovery from eccrine sweat was evaluated for the first time in a clinical proof-of-concept study with respect to predictive diagnostics, targeted prevention and personalisation of medical services. Nine breast cancer patients were monitored by assessing metabolites in eccrine sweat at four timepoints during the first cycle of first-line neoadjuvant chemotherapy. Patients of the Luminal B, HER2-positive Luminal and Triple-Negative subtypes were included for whom chemotherapy was mostly based on a cytotoxic combination of epirubicin and cyclophosphamide (EC). Sweat collection was assisted by nurses and was well accepted by the patients. This study reports a first description of the comprehensive metabolic profile of eccrine sweat in nine breast cancer patients. Metabolites were detected over four orders of magnitude, and among the metabolites with highest intensity in eccrine sweat, we identified urocanic acid, pyroglutamic acid, choline and the amino acids serine, threonine, aspartate and valine (Fig. 2a). Particularly, urocanic acid was previously characterized as a metabolite specific of the interstitial fluid [57], but may also be found in skin. By transforming intensity values of metabolites identified in matched eccrine sweat and blood plasma samples in a scatter plot to a similar scale (Fig. 2b), we demonstrated that metabolites did not scatter randomly. Asp and Gln were preferentially found in eccrine sweat and blood plasma, respectively, as expected by their roles in metabolism. Gln is required for anabolism, and Asp may act as a natural moisturizing agent. This is an indication that eccrine sweat is a regulated compartment subject to specific homeostatic control.

Small molecule therapeutics were detected in eccrine sweat of breast cancer patients (Fig. 3). Cyclophosphamide was a component of the anticancer therapy, and ondansetron was a component of the simultaneously administered anti-emetic therapy. Furthermore, the time-dependent intensity changes of these agents correctly mirrored the administration schedule, underlining the possibility to assess individual dynamic biomarker profiles in breast cancer patients. Although the CVs of metabolites from eccrine sweat were generally large, the effect sizes of these drugs still enable determining statistically significant regulations. The larger variation of metabolites in eccrine sweat compared to plasma indicates that improvements in normalization strategies are necessary to increase the precision of this approach, e.g. by accounting for individual sweat rate. Ondansetron was not detected in eccrine sweat at baseline, but in each patient 2 h after starting the administration (t_1_). While ondansetron levels returned to baseline in six patients at recovery (t_3_), they remained prominent in patients P02, P03 and P08, although all patients received the same amount of ondansetron. These differences did not correlate with the time span between the last scheduled ondansetron intake and the recovery visit (t_3_) and might be due to individual clearance mechanisms from the interstitial space. Cyclophosphamide was also detected in the same time-dependent pattern with increased intensity during the administration phase (t_1_ and t_2_). However, this anticancer agent remained undetectable in P01 and P10 during the administration phase, consistent with the treatment plan as they did not receive cyclophosphamide (Fig. 3c). These findings underscore the potential of eccrine sweat for individualised drug monitoring and may improve the personalisation of medical services in the context of 3PM.

Eccrine sweat originates from the interstitial fluid [44], but there is only scarcely available literature about drug distribution between plasma and the interstitium [57, 59, 60]. Strikingly, although only a small number of metabolites was found significantly regulated, several were significantly regulated in both eccrine sweat and blood plasma, including the non-essential amino acids asparagine, serine and proline, as well as ornithine (Fig. 4). While they continuously decreased in blood plasma over the treatment cycle, the same metabolites typically showed increased levels in eccrine sweat at recovery compared with the administration phase. The present observation that the same metabolites are regulated differently in eccrine sweat and plasma may have important implications for monitoring disease progression in breast cancer patients and thus targeted prevention. Therefore, metabolites in eccrine sweat represent systemic properties of the patient metabolism that are inaccessible by plasma analysis. While to the best of our knowledge, there is no literature evidence that FEC-based chemotherapy would directly affect these amino acids, their down-regulation in plasma may be interpreted as a consequence of lower systemic mobilization of these building blocks by the tumour. Indeed, amino acid depletion to support anticancer therapy was found useful in some studies [61], especially with arginine [62], asparagine and methionine [63]. In combination with the eccrine sweat data, however, the observed down-regulation of those amino acids in plasma may rather be explained as a phenomenon to sustain interstitial fluid levels at the cost of plasma levels.

Since first-line therapy in breast cancer patients of this pilot study relied on DNA-damaging agents with a slower onset compared with the sampling schedule at t_0_–t_2_, we expected only minor immediate systemic effects at these time points. This was corroborated by a plasma multi-omics analysis including proteomics, fatty acids/oxylipins and targeted metabolomics. Accordingly, we found only three significantly regulated proteins in blood plasma, including HBD, ACTB and gelsolin, indicating cytotoxic treatment effects [64]. We also found minor regulations on the level of the fatty acids and oxylipins in plasma. This was most probably related to the administration of dexamethasone, which was used as an anti-emetic and immune suppressant. Dexamethasone inhibits phospholipase A2 activity [65] and thereby suppresses the formation of oxylipins. Furthermore, dexamethasone is known to reduce blood cortisol levels [66]. Cortisol showed a pronounced down-regulation in plasma during the administration phase compared to baseline, except in patient P01, who featured already low cortisol levels at t_0_ (Fig. 4). The individual treatment schedule revealed that this patient received dexamethasone already on the day before the anticancer therapy. Therefore, the reduction of cortisol in plasma seems to reflect a direct impact of dexamethasone in this study. The cholesteryl esters CE18:2 and CE 20:4 were also significantly down-regulated in plasma over the course of the first cycle of chemotherapy. Intratumour cholesteryl esters were previously associated with cell proliferation in breast cancer [67]. A tumour-associated formation of cholesteryl esters in the breast cancer patients was indicated by downregulated cholesterol levels at recovery compared to baseline in the clinical parameters (Supplementary Fig. S3c). The plasma multi-omics analysis confirmed the systemic impact of cytotoxic chemotherapy in combination with phospholipase A2 inhibition. This informed the interpretation of the eccrine sweat metabolites in this study, especially with respect to treatment effects.

RCB is a multi-parameter prognostic factor to estimate long-term survival in breast cancer patients [18]. The RCB score is established after neoadjuvant chemotherapy, followed by collecting information about the primary tumour and lymph nodes. Patients of this study presented with RCB classes of 0–III (Table 1). They were stratified into RCB low (class 0–I, *n* = 4) and RCB high (class II–III, *n* = 5) groups and were comparatively evaluated at baseline and recovery (Fig. 5). RCB stratification did not reveal a significant impact on the protein or fatty acid/oxylipin levels. Eccrine sweat, however, showed a pronounced metabolic signature associated with low amino acid and amino acid-related metabolites at baseline for the RCB high group. Largely, the same metabolites were identified at recovery, albeit most were not significantly regulated anymore. In contrast to the evaluation of the systemic effects, where non-essential amino acids were significantly regulated, RCB stratification showed essential amino acids specific to the RCB low group. Therefore, eccrine sweat promises to improve predictive diagnostics by stratifying patient groups according to distinct metabolic states that in turn depend on the systemic contribution of breast cancer. The platelet-derived 12-HHTrE was the only oxylipin upregulated at the recovery timepoint in RCB high (Fig. 5b). 12-HHTrE was already found elevated in breast cancer patients [68] and is implicated in modulating haemostasis [69]. Again, this potentially indicates off-target effects of the cytotoxic therapy on haemopoiesis.

Eccrine sweat also revealed individual lifestyle parameters that may support risk assessments. For example, patient P01 was a declared smoker and displayed elevated intensities of nicotine and cotinine simultaneously, the latter being the primary metabolite of nicotine. The identification of the nicotine metabolite cotinine is crucial to categorize smoker from non-smokers or passive smokers. The latter may be shown by the occasional finding of nicotine and cotinine over the treatment period (Supplementary Fig. S5). Simple surface contamination is indicated by the detection of nicotine alone without metabolites. The evidence-based assessment of lifestyle parameters from eccrine sweat represents a promising application of eccrine sweat in the clinical context.

## Limitations

Metabolic analysis of eccrine sweat using mass spectrometry is an emerging technology. It is based on a non-invasive sample collection and enables metabolic human biomonitoring. Mass spectrometry represents a powerful method to broadly assess complex metabolite profiles from eccrine sweat. Despite the positive results obtained in this pilot study, the metabolic patterns related to metabolic turnover in breast cancer patients as a means for stratification have to be verified in a larger prospective study, which is currently planned. Such a study may also include more diverse patient populations. Additionally, sweat collection may be associated with considerable variation, which needs to be accounted for during data interpretation and cohort design. However, dynamic changes in metabolites in eccrine sweat are typically large, resulting in appropriate effect sizes for scientific investigation. Further work will allow to improve the experimental precision and power of this novel approach. This study was performed using an untargeted metabolomics approach to determine comprehensive metabolic profiles from eccrine sweat, but targeted assays focussing on specific metabolite panels will need to be established for implementation in clinical laboratories.

## Conclusions and expert recommendations in the context of 3PM

This study provides first evidence that metabolic biomarkers of breast cancer patients can be determined from eccrine sweat. Eccrine sweat collection is completely non-invasive, risk-free, cost-efficient and was well accepted by the patients. Eccrine sweat enables assessing comprehensive metabolic profiles. Its content is surprisingly diverse, amounting to hundreds of exogenous and endogenous metabolites. In the course of this pilot study, breast cancer patients received first-line therapy and were monitored by collecting eccrine sweat over the course of the first therapy cycle from baseline to recovery. We are convinced that the metabolic analysis of eccrine sweat will support 3PM aspects in this context by contributing to predictive diagnostics, targeted prevention and personalisation of medical service.

### Contribution to predictive diagnostics

Patient stratification is an essential component of predictive diagnostics. Metabolic patterns in eccrine sweat related to metabolic turnover at baseline correlated with RCB in breast cancer patients and may thus enable patient stratification. This metabolic pattern was mostly driven by essential amino acids. Altered metabolic turnover in breast cancer patients may be a proxy for the extent of the systemic contribution of disease, which may explain the correlation with RCB. The presented discovery will be verified in an upcoming study of our laboratory with improved power. Additionally, the metabolic analysis of eccrine sweat is a powerful tool to detect lifestyle parameters that may inform risk assessments and, therefore, may be implemented in predictive diagnostics of breast cancer. For example, a separation of smokers and non-smokers can be achieved by the simultaneous detection of nicotine and cotinine, which indicates direct exposure through inhalation and excludes surface contamination.

### Contribution to personalization of medical services

The qualitative and quantitative analysis of small molecules in the eccrine sweat of breast cancer patients has successfully enabled individual monitoring of the therapy. Specifically, cyclophosphamide and ondansetron were successfully detected and monitored over the course of the first treatment cycle and correctly reflected individual treatments. Importantly, the dynamic changes of these small molecules covered three orders of magnitude. The return from peak to baseline abundance of small molecule therapies in eccrine sweat after the treatment may reflect individual clearance rate from the interstitial space.

### Contribution to targeted prevention

Eccrine sweat originates from the interstitial fluid [44]. However, the exact mechanisms of distribution from blood plasma to the interstitium and subsequent secretion from eccrine glands remain largely speculative [38]. The interstitial fluid is usually challenging to access, but is of high medical importance because it represents the site of action of most therapeutics and may represent a surrogate for the systemic impact not only of breast cancer. Interestingly, we observed distinct regulations of non-essential amino acids over the course of the treatment cycle in eccrine sweat and blood plasma of breast cancer patients, which indicated that these compartments are indeed under separate homeostatic control. This potentially opens up a new dimension for biomonitoring of breast cancer in secondary and tertiary care. Since eccrine sweat collection does not require trained personnel and may be performed in a decentralised manner, longitudinal monitoring may be realised and inform about disease stabilization or progression in individual patients.

### Outlook in the framework of 3PM

Comprehensive metabolic profiles of eccrine sweat show potential for broad implementation in secondary and tertiary care of breast cancer patients in the context of 3PM. Further work will aim to control precision through improved normalization strategies, e.*g*. by accounting for individual sweat rates. In addition, the unprecedented ability to simultaneously capture endogenous metabolites and exogenous lifestyle parameters could enable investigations into how lifestyle parameters influence drug efficacy or disease progression. The simple and non-invasive sample collection for the individualised assessment of health status and drug exposure by metabolic biomarker analysis from eccrine sweat will be a key factor to advance 3PM concepts in daily practice [1, 5, 37]. Specifically, metabolic analysis of eccrine sweat is expected to significantly advance evidence-based predictive diagnostics, targeted prevention, including patient stratification, and personalised treatment options even beyond secondary and tertiary care of breast cancer.

## Supplementary Information

Below is the link to the electronic supplementary material.

ESM1 (DOCX 1.40 MB)

ESM2 (XLSX 10.5 KB)

ESM3 (XLSX 15.9 KB)

ESM4 (CSV 129 KB)

ESM5 (XLSX 50.2 KB)

ESM6 (XLSX 128 KB)

## Data Availability

All anonymized data associated with this study are presented in the paper or the article supplementary information and supplementary data. Any additional information may be requested from the corresponding author.
