# Supplementary material for: Mass spectrometry-based analysis of eccrine sweat supports predictive, preventive and personalised medicine in a cohort of breast cancer patients in Austria
Source: EPMA J. 2025 Jan 31;16(1):165–82. doi: 10.1007/s13167-025-00396-6 (PMC11842658; doi:10.1007/s13167-025-00396-6)
Supplement: Supplementary file 1 — (DOCX 1.40 MB) [file 13167_2025_396_MOESM1_ESM.docx]

Supporting Information to

**Mass spectrometry-based analysis of eccrine sweat supports predictive, preventive and personalised medicine in a cohort of breast cancer patients in Austria**

Michael Bolliger, Daniel Wasinger, Julia Brunmair, Gerhard Hagn, Michael Wolf, Karin Preindl, Birgit Reiter, Andrea Bileck, Christopher Gerner, Florian Fitzal, Samuel M. Meier-Menches


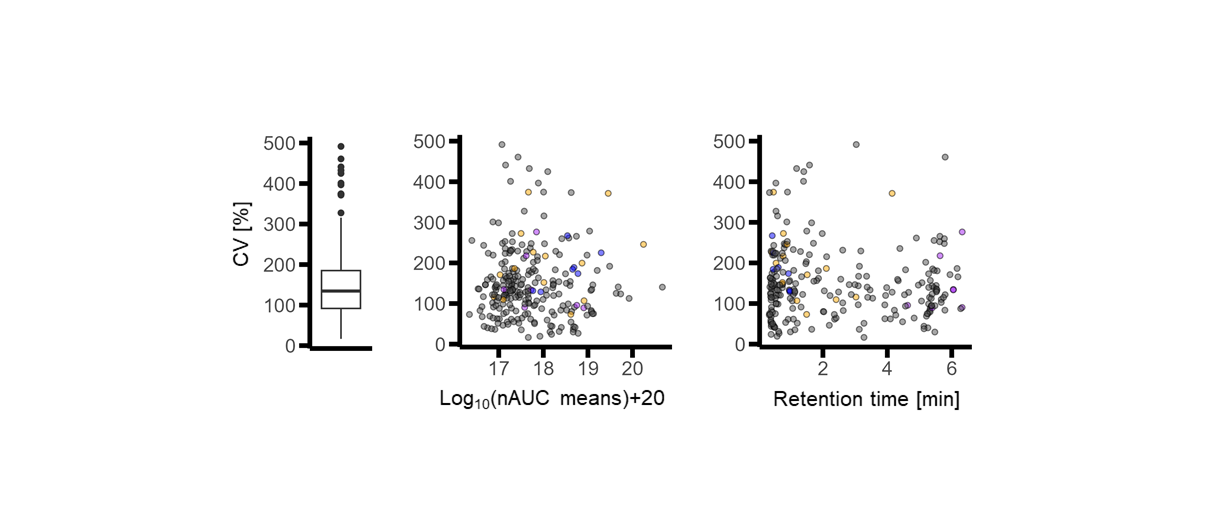


**Supplementary Figure S1**. (*left*) The box plot shows the distribution of the coefficients of variation (CVs) of the 247 metabolites identified across 36 samples. Normalized area under the curve values were used for CV calculation. The scatter plots show that CVs are largely independent of intensity (*center*) and retention time (*right*). The colour code indicates amino acid(-related) (yellow), lipid related (purple) and nucleotide related (blue) metabolites, including N-AcGlA = N-acetylglucosamine.


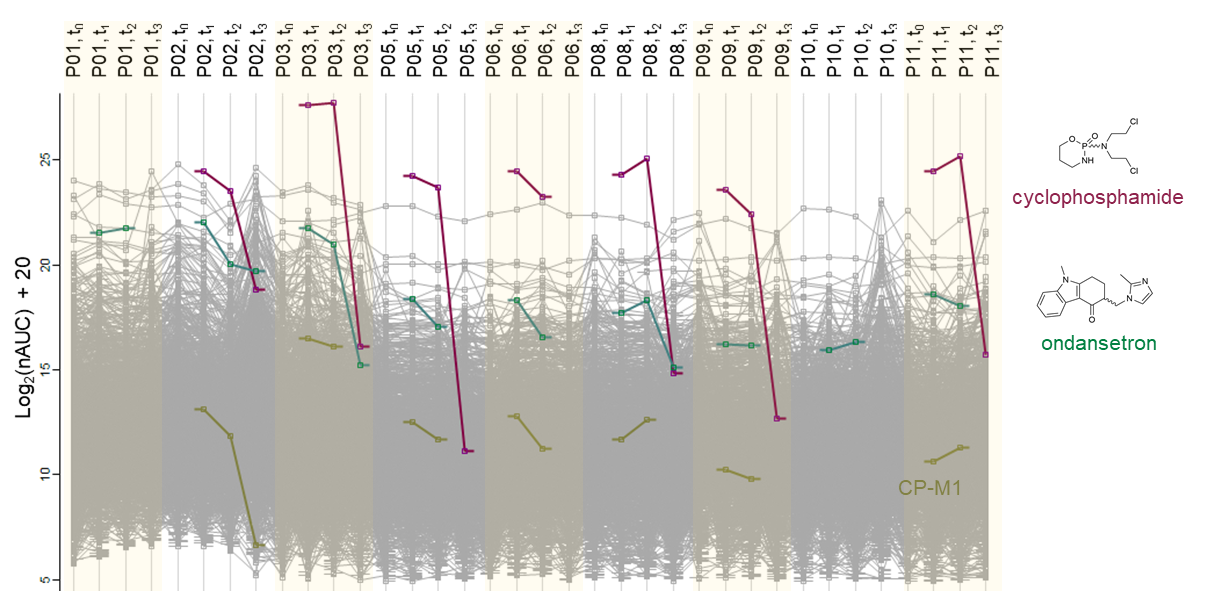


**Supplementary Figure S2**. Profile plot of Log2-transformed and normalized areas under the curve of metabolites of all eccrine sweat samples of patients P01–P11 at the four sampling time points, corresponding to baseline (t_0_), 2h after start of the infusion (t_1_), after infusion completion (t_2_) and at recovery (t_3_). The profiles contain missing values. The highlighted metabolites refer to cyclophosphamide, ondansetron and CP-M1.


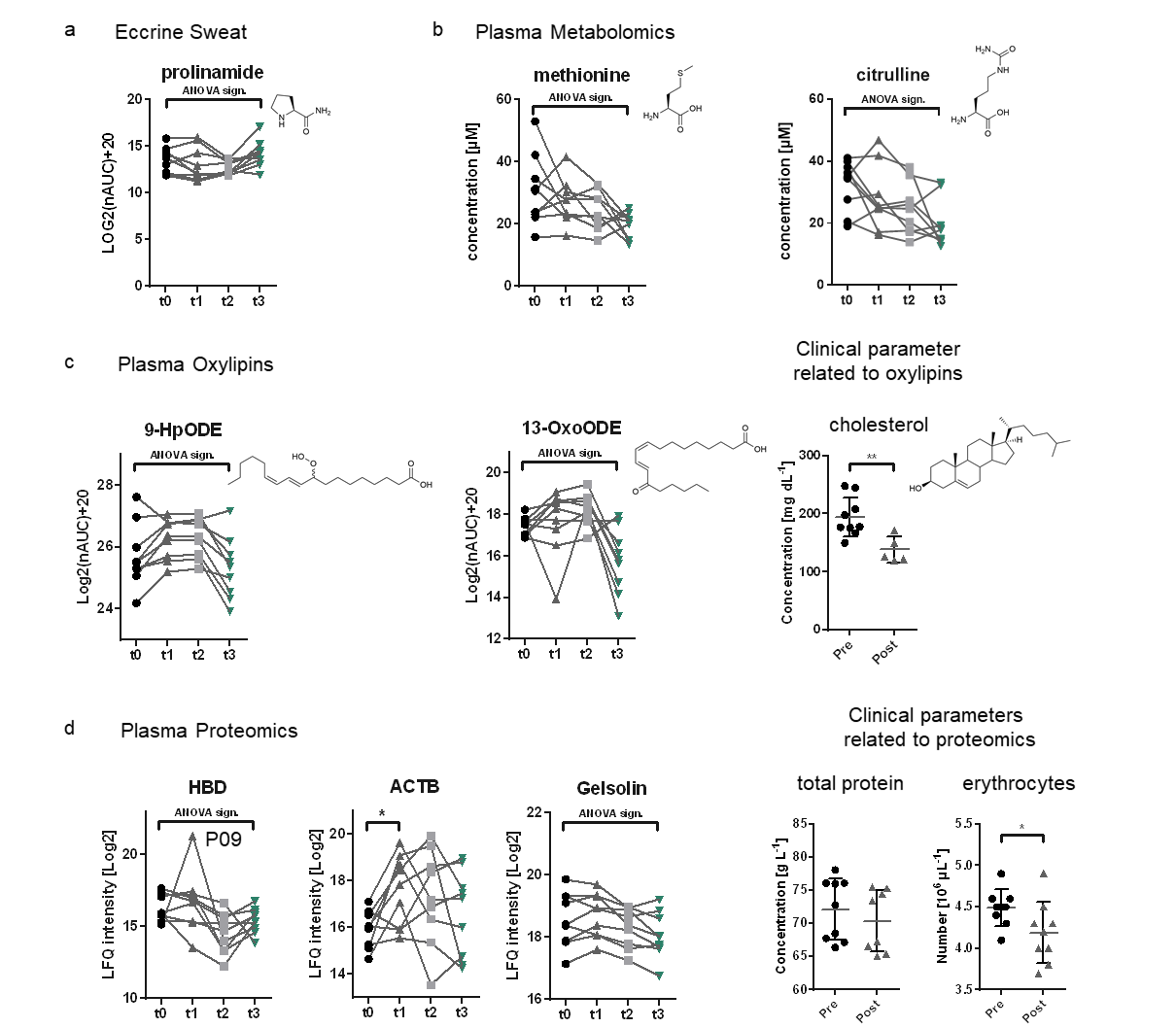


**Supplementary Figure S3**. Effect of therapy on eccrine sweat and plasma metabolites in breast cancer patients. ANOVA-testing revealed therapy-related and ANOVA-significantly regulated metabolites in eccrine sweat (**a**) and plasma, including metabolites (**b**), oxylipins (**c**) and proteins (**d**) with associated clinical parameters.


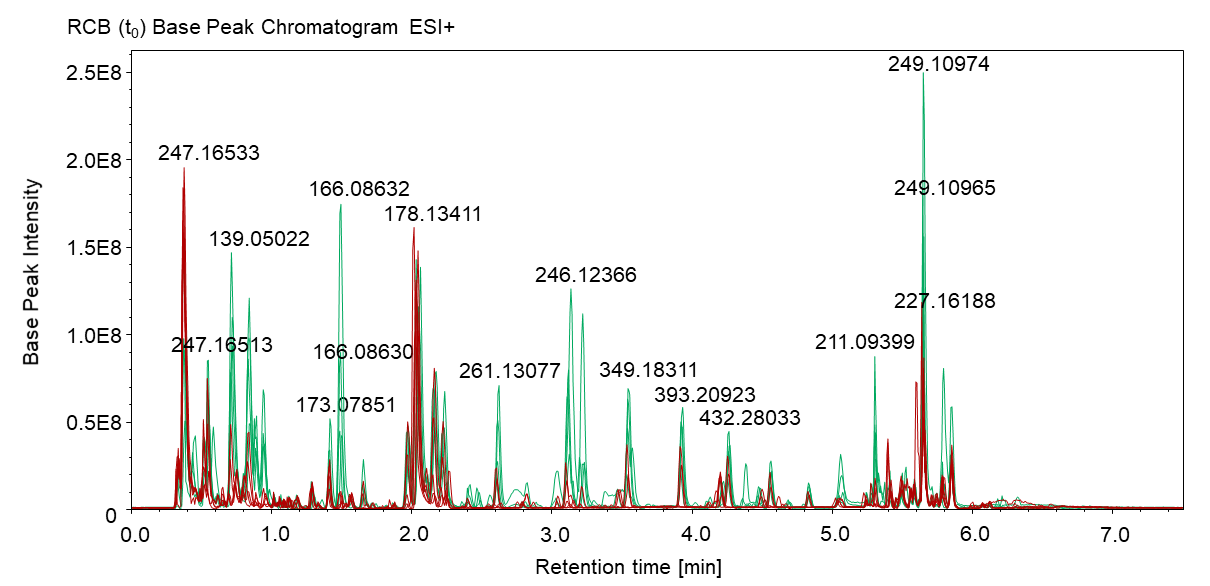


**Supplementary Figure S4**. Comparison of the metabolic differences according to the two RCB groups (low = green, high = red) at baseline in eccrine sweat via summed extracted ion chromatograms of individual patients.


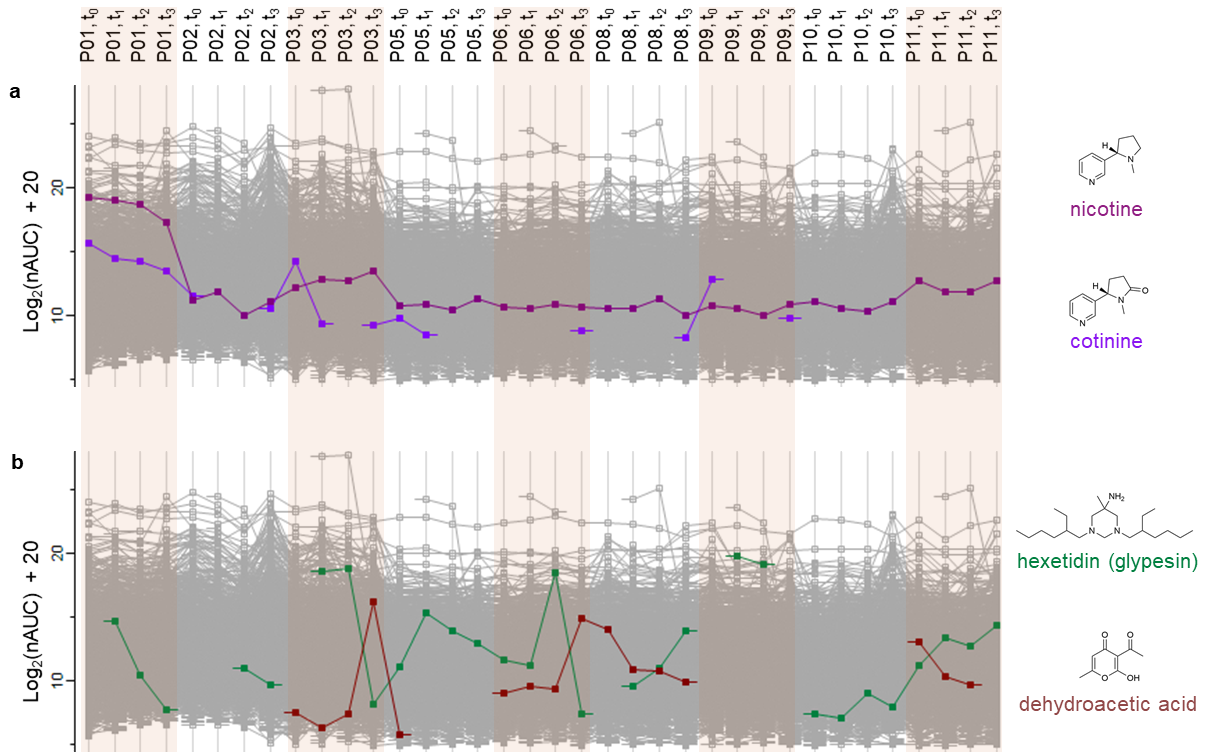


**Supplementary Figure S5**. Profile plot of Log2-transformed and normalized areas under the curve of metabolites of all eccrine sweat samples of patients P01–P11 at the four sampling time points, corresponding to baseline (t_0_), 2h after start of the infusion (t_1_), after infusion completion (t_2_) and at recovery (t_3_). The profiles contain missing values. The profile plots show lifestyle parameters associated with smoking (**a**) and application of disinfectants (**b**).

**Supplementary Table S1**. List of internal standards used for oxylipin analysis.

| **Internal standard** | **Abbreviation** | **Conc. [pg/µL]** |
| --- | --- | --- |
| 12S-hydroxyeicosatetraenoic acid-d8 | 12S-HETE-d8 | 6,67 |
| 15S-hydroxyeicosatetraenoic acid-d8 | 15S-HETE-d8 | 6,67 |
| 5-oxo-eicosatetraenoic acid-d7 | 5-OxoETE-d7 | 20 |
| 11,12-dihydroxy-5Z,8Z,14Z-eicosatrienoic acid-d11 | 11,12-DiHETrE-d11 | 6,67 |
| prostaglandin E2-d4 | PGE2-d4 | 13,33 |
| 20-hydroxyeicosatetraenoic acid-d6 | 20-HETE-d6 | 6,67 |

**Supplementary Table S2**. Inclusion list covering 33 *m/z*-values specific for oxylipins and oxylipin precursor molecules.

| ***m/z*-value** | ***m/z*-value** | ***m/z*-value** | ***m/z*-value** |
| --- | --- | --- | --- |
| 254,2245 | 311,2228 | 333,2071 | 359,2222 |
| 275,2011 | 313,2384 | 335,2222 | 367,3576 |
| 277,2167 | 315,1966 | 337,2384 | 375,2171 |
| 279,2324 | 317,2122 | 343,2279 |  |
| 281,2480 | 319,2279 | 348,3069 |  |
| 283,2637 | 321,2435 | 349,2020 |  |
| 293,2122 | 325,2382 | 351,2177 |  |
| 295,2279 | 327,2324 | 353,2328 |  |
| 301,2168 | 327,2781 | 355,2428 |  |
| 303,2324 | 329,2480 | 357,2585 |  |

**Supplementary Table S3**. Degree of identification of oxylipins and other lipids identified in plasma.

| **COMPOUND** | **DEGREE OF IDENTIFICATION** |
| --- | --- |
| 11-HDoHE | Exact mass, retention time and MS2 + standard |
| 11-HETE | Exact mass, retention time and MS2 + standard |
| 12-HEPE | Exact mass, retention time and MS2 + standard |
| 12-HETE / 8-HETE | Exact mass, retention time and MS2 + standard |
| 12-HETrE | Exact mass, retention time and MS2 |
| 12-HHTrE | Exact mass, retention time and MS2 + standard |
| 13,14-dihydro-15-keto-PGE2 | Exact mass, retention time and MS2 + standard |
| 13,14-dihydro-15-keto-PGF2a | Exact mass, retention time and MS2 + standard |
| 13-,16- and 17-HDoHE | Exact mass, retention time and MS2 + standard |
| 14-HDoHE and 10-HDoHE | Exact mass, retention time and MS2 + standard |
| 15-HETE | Exact mass, retention time and MS2 + standard |
| 313_10.75 | Exact mass, retention time |
| 317_10.65 | Exact mass, retention time |
| 335_9.54 | Exact mass, retention time |
| 9- and 13-HODE_9(10)- and 12(13)-EPOME | Exact mass, retention time and MS2 + standard |
| 9-HOTrE | Exact mass, retention time and MS2 |
| 9-HpODE | Exact mass, retention time and MS2 + standard |
| Hepoxilin B3 | Exact mass, retention time and MS2 |
| HHTrE / isoform I | Exact mass, retention time and MS2 |
| HHTrE / isoform II | Exact mass, retention time and MS2 |
| HpODE / isoform I | Exact mass, retention time and MS2 |
| LPC (0:0/16:0) | Exact mass, retention time and MS2 |
| LPC (0:0/18:1) | Exact mass, retention time and MS2 |
| LPC (0:0/18:2) | Exact mass, retention time and MS2 |
| LPC (0:0/20:4) | Exact mass, retention time and MS2 |
| LPC (0:0/22:6) | Exact mass, retention time and MS2 |
| LPC (16:0/0:0) | Exact mass, retention time and MS2 |
| LPC (18:1/0:0) | Exact mass, retention time and MS2 |
| LPC (18:2/0:0) | Exact mass, retention time and MS2 |
| LPC (20:4/0:0) | Exact mass, retention time and MS2 |
| LPC (22:6/0:0) | Exact mass, retention time and MS2 |
| LPE (0:0/16:0) | Exact mass, retention time and MS2 |
| LPE (0:0/18:1) | Exact mass, retention time and MS2 |
| LPE (0:0/18:2) | Exact mass, retention time and MS2 |
| LPE (0:0/20:4) | Exact mass, retention time and MS2 |
| LPE (0:0/20:5) | Exact mass, retention time and MS2 |
| LPE (0:0/22:6) | Exact mass, retention time and MS2 |
| LPE (16:0/0:0) | Exact mass, retention time and MS2 |
| LPE (18:1/0:0) | Exact mass, retention time and MS2 |
| LPE (18:2/0:0) | Exact mass, retention time and MS2 |
| LPE (20:4/0:0) | Exact mass, retention time and MS2 |
| LPE (22:6/0:0) | Exact mass, retention time and MS2 |
| PGE2 | Exact mass, retention time and MS2 + standard |
| PGF2a | Exact mass, retention time and MS2 + standard |
| Sphingosine-1-phosphate | Exact mass, retention time and MS2 |
| TXB2 | Exact mass, retention time and MS2 + standard |
